# Stable plastid transformation in kiwifruit (*Actinidia chinensis*)

**DOI:** 10.1007/s42994-024-00186-0

**Published:** 2024-11-28

**Authors:** Qiqi Chen, Yuyong Wu, Yanchang Wang, Jiang Zhang, Shengchun Li

**Affiliations:** 1 Key Laboratory of Plant Germplasm Enhancement and Specialty Agriculture, Wuhan Botanical Garden, Chinese Academy of Sciences, Wuhan, 430074 China; 2 State Key Laboratory of Biocatalysis and Enzyme Engineering, School of Life Sciences, Hubei University, Wuhan, 430062 China; 3 School of Pharmacy, Binzhou Medical University, Yantai, 264003 China; 4 Shenzhen Branch, Guangdong Laboratory of Lingnan Modern Agriculture, Key Laboratory of Synthetic Biology, Ministry of Agriculture and Rural Affairs, Agricultural Genomics Institute at Shenzhen, Chinese Academy of Agricultural Sciences, Shenzhen, 518000 China

**Keywords:** *Actinidia chinensis*, Woody vine, Transplastomic technology, *aadA*, Green fluorescent protein

## Abstract

Plastid transformation offers valuable benefits in plant biotechnology, such as high-level transgene expression and the absence of gene silencing. Here we describe the first protocol of a plastid transformation system for a woody vine (liana) kiwifruit (*Actinidia chinensis*). The transgenic DNA carries a spectinomycin-resistance gene (*aadA*) cassette and a green fluorescent protein (*GFP*) reporter gene cassette, flanked by two adjacent kiwifruit plastid genome sequences, thereby allowing targeted insertion between the *trnfM* and *trnG* genes. Six spectinomycin-resistant shoots were obtained out of 12 plates subjected to bombardment, and two were positive events, confirmed through PCR and Southern blot analyses. The GFP was localized to plastids as monitored by confocal laser scanning microscopy and reached 2.5% of leaf total soluble protein. Success in kiwifruit extends transplastomic technology of woody species beyond poplar, and will provide an attractive biosynthetic chassis for molecular farming.

**Supplementary Information:**

The online version contains supplementary material available at 10.1007/s42994-024-00186-0.

## Introduction

Transformation of the plastid genome offers multiple advantages over conventional nuclear transformation, including potential for extremely high levels of transgene expression (Castiglia et al. 2016; Oey et al. 2009), accommodation of the transgene into the plastid genome, through homologous recombination without position effects (Bock 2015), and the possibility of stacking multiple transgenes in synthetic operons (Scharff and Bock 2014; Yang et al. 2022). Successful plastid transformation was first established in a unicellular alga, *Chlamydomonas reinhardtii* (Boynton et al. 1988), and two years later in a seed plant, tobacco (Svab et al. 1990). Over the past 30 years, transplastomic technology was further extended to more than 20 seed plants (Liu et al. 2023). However, major crops, such as rice, wheat and maize, were missing in the transformable list, and poplar was the only woody species.

Kiwifruit, belonging to the genus *Actinidia* that originated in China, comprises approx. 54 species (Yue et al. 2020). It is well known as the “king of fruits”, owing to its extremely high content of vitamin C, nutritional minerals, and diverse metabolites that are beneficial for human healthy (Li et al. 2023). *Agrobacterium*-mediated nuclear transformation in kiwifruit was first achieved in 1991 and became routine in multiple species (Uematsu et al. 1991; Wang et al. 2006; Wang and Wang 2007; Yao et al. 2023). Kiwifruit was genetically modified for abiotic and biotic stress tolerance (Nakamura et al. 1999; Tian et al. 2011), and quality improvement (Bulley et al. 2012; Kim et al. 2010; Peng et al. 2020). Unlike most other plants, in which the plastid genome is maternally inherited, the kiwifruit exhibits a complex system of plastid inheritance, with possible transmission through both maternal and paternal lines (Li et al. 2013).

Development of kiwifruit plastid transformation is still attractive for efficient production of edible vaccines, biopharmaceuticals, and antibodies, due to the high-level accumulation of recombinant protein that can be generally achieved in transplastomic plants (up to 75% of the total soluble protein) (Castiglia et al. 2016). In addition, plastid-transformed kiwifruit would provide a useful tool to study the complex inheritance patterns of the plastid in kiwifruit. In this study, we present an efficient plastid transformation protocol for kiwifruit (*Actinidia chinensis* cv. ‘Hongyang’). The establishment of transplastomic technology will open the door for new synthetic biology applications in kiwifruit plastids.

## Results

### Optimization of a plant regeneration and selection system for kiwifruit

To develop an efficient leaf-based regeneration and selection regime for kiwifruit, we first searched for an appropriate medium composition for regeneration. Small pieces of leaf explants (5 × 5 mm) were placed on five different regeneration media, herein referred to as AcReMs (Table S1), to determine the optimal medium for regeneration from young leaf explants. After 60 days in culture, green calli were induced on all the AcReMs, and the maximum number of shoots was obtained on AcReM3, which contained a combination of 1 mg/L thidiazuron (TDZ), 2 mg/L 6-benzyladenine (6-BA) and 1 mg/L α-naphthalene acetic acid (NAA).

For selection of plastid transformed kiwifruit, we used the spectinomycin-resistance gene *aadA* as a selectable marker, which has previously been successfully utilized for plastid transformation of many plant species (Bock 2015; Liu et al. 2023). To determine the appropriate concentration for selection, leaf explants were cultivated on AcReM3 supplemented with various concentrations of spectinomycin (0, 100, 150, 200, 250 and 300 mg/L). After 60 days of selection, partial green calli were observed on AcReM3 containing spectinomycin at less than 150 mg/L, whereas leaf explants were completely bleached when exposed to 200 mg/L spectinomycin (Fig. S1A). To effectively suppress background growth (Ruf et al. 2019), a higher spectinomycin concentration of 300 mg/L was used for further selection of the kiwifruit transplastomes.

For the optimization of biolistic DNA delivery parameters, a nuclear transformation vector containing the β-glucuronidase (*GUS*) gene cassette was employed. Based on transient *GUS* expression results, it was established that 1100 psi rupture disk was optimal for DNA delivery, when the target distance was kept constant at 6 cm (Fig. S1B). Further studies on the effect of target distance indicated that highest *GUS* expression was at 9 cm (Fig. S1C). Therefore, leaf explants for plastid transformation were bombarded with 1100 psi at a distance of 9 cm.

### Construction of kiwifruit plastid transformation vector

**Fig. 1:**
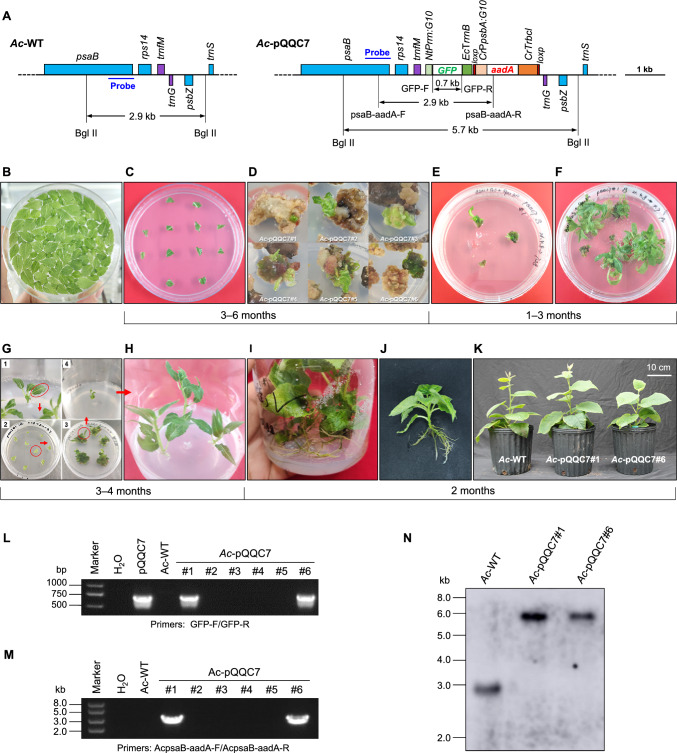
Generation of plastid-transformed kiwifruit. **A** Physical maps of the targeting region in the kiwifruit plastid genome (ptDNA, left) and the plastid transformation vector pQQC7. *Nt*P*rrn*: plastid 16S rRNA promoter from *Nicotiana tabacum*; *Cr*P*psaA*: *psaA* promoter from *Chlamydomonas reinhardtii*; *T7g10*: 5′ UTR of *gene10* from bacteriophage T7. **B** Preparation of kiwifruit leaves for particle bombardment. **C** Bombarded leaf explants were exposed to AcReM3 containing spectinomycin. Spectinomycin was used to select for transplastomic lines. **D** Spectinomycin-resistant calli appeared after three months on selection medium, indicating successful plastid transformation. **E**, **F** These calli were able to grow into shoots. **G**, **H** Leaves of these lines were subjected to additional rounds of regeneration in order to achieve homoplasmy. **I**, **J** Progression of shoot growth and root induction of transplastomic lines (*Ac*-pQQC7). Transplastomic lines showed normal shoot growth and development of roots. A timeline illustrating the estimated approximate duration of the individual steps in the protocol is given below. **K** Growth comparison between transplastomic *Ac*-pQQC7 and wild-type (*Ac-*WT) plants under greenhouse conditions. Transplastomic plants exhibited similar growth patterns to wild-type plants. **L**, **M** PCR amplification using *GFP*-specific primes and psaB-aadA-F/psaB-aadA, which yielded 720 bp (**L**) and 2.9 kb (**M**) amplicons, respectively, confirmed the presence of the transgene in *Ac*-pQQC7 plants. **N** Southern blot analysis verified the homoplasmy of *Ac*-pQQC7. A ~ 5.7 kb signal was observed in *Ac*-pQQC7, whereas the untransformed plants showed a 2.9 kb band on hybridization with the *psaB* probe

To construct the kiwifruit plastid transformation vector, pQQC7, we amplified species-specific flanking sequences from kiwifruit genomic DNA (Fig. S2). In the pQQC7 vector, the marker gene *GFP* (Green fluorescent protein) was driven by the tobacco plastid 16S rRNA promoter, which was fused with the 5′ untranslated region (UTR) from *gene10* of bacteriophage T7 (*Nt*P*rrn*:*T7g10*). The selectable marker gene *aadA*, which confers resistance to spectinomycin, was controlled by the *psbA* promoter from *Chlamydomonas reinhardtii*, in combination with the 5′ UTR from *T7g10* (*Cr*P*psbA*:*T7g10*). The *GFP* and *aadA* cassettes were integrated into the *trnfM*/*trnG* intergenic region of the kiwifruit plastid genome (Fig. 1A, Fig. S2).

### Production and analyses of transplastomic kiwifruit plants

After being placed on an osmotic medium (AcOsM) in the dark overnight (Fig. 1B), sterile kiwifruit leaves were bombarded with plasmid pQQC7 using a 1100 psi rupture disk at a target distance of 9 cm. Primary spectinomycin-resistant green calli began to appear after three months of incubation of bombarded leaf explants on AcReM3 including 300 mg/L spectinomycin (Fig. 1C). Subsequently, primary spectinomycin-resistant calli began to appear after three months. After six months under selection, six green calli were obtained from 12 plates (Fig. 1D). Six independent transplastomic lines (*Ac*-pQQC7) underwent elongation and multiplication on shoot multiplication medium (AcSmM) containing 300 mg/L spectinomycin (Fig. 1E, F). To achieve homoplasmy, the young leaves of these transplastomic lines underwent additional rounds of regeneration (Fig. 1G, H). After root induction (Fig. 1I, J), the transplastomic lines were transferred to soil and did not exhibit any discernible phenotypic difference when compared with the wild type (Fig. 1K).

To confirm the presence of the transgene in the shoots, we performed PCR using specific primers (GFP-F/GFP-R) designed for the *GFP* gene. This resulted in the amplification of a 720-bp PCR product (Fig. 1L), indicating the presence of the *GFP* gene. Moreover, we designed a primer pair (psaB-aadA-F/psaB-aadA-R) to target the *psaB* region of the native chloroplast genome and the *aadA* marker, respectively. PCR amplification with these primers yielded a 2.9 kb product (Fig. 1M). Two PCR-positive lines (*Ac*-pQQC7#1, #6) were confirmed to have reached homoplasmy through Southern blot analysis. In the wild-type plants (*Ac*-WT), a 2.9 kb fragment was detected, whereas in the *Ac*-pQQC7, a 5.7 kb fragment was observed corresponding to the integration of the transgene (Fig. 1N).

### Determination of GFP expression levels in transplastomic plants

**Fig. 2:**
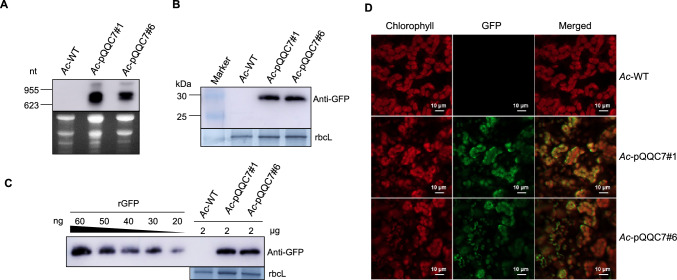
Analysis of *GFP* expression in transplastomic kiwifruit plants. **A** Northern blot analysis of the *GFP* transcripts. **B** Western blot analysis confirmed the accumulation of GFP in *Ac*-pQQC7 leaves using an anti-GFP antibody. The larger bands are likely the results of read-through transcripts owing to inefficient transcription termination in plastids (Lu et al. 2017; Zhou et al. 2007). **C** Semi-quantitative analysis of GFP accumulation in *Ac*-pQQC7 using a dilution series of recombinant GFP (rGFP). **D** Verificaiton of plastid *GFP* expression in leaf cells using confocal laser-scanning microscopy

To examine *GFP* expression, Northern blot analysis was performed using a hybridization probe specific for the *GFP* coding region. The blots revealed two transcripts, with the smaller and more abundant transcript representing the expected full-length *GFP* mRNA (Fig. 2A). To determine the accumulation level of GFP, we conducted Western blot analysis using an anti-GFP antibody and a dilution series of recombinant GFP as a reference. The anti-GFP antibody successfully detected a 27 kDa GFP peptide, confirming GFP production in the transplastomic lines (Fig. 2B). Based on our estimation, GFP accumulation reached approx. 2.5% of the total soluble protein (Fig. 2C). Furthermore, the presence of GFP fluorescence, specifically within the chloroplasts, confirmed its confinement to the chloroplast compartment in the leaves of the *Ac*-pQQC7 lines (Fig. 2D).

## Discussion

This study presents the first successful protocol for generating stable transplastomic kiwifruit plants. In our plastid transformation system, the transgene was targeted between the *trnG* and *trnfM* genes of the kiwifruit plastid genome (Fig. 1A). In the transplastomic kiwifruit, the homoplastomic state was verified by Southern blot analysis (Fig. 1N). Furthermore, northern blot and Western blot analyses confirmed expression of reporter gene (Fig. 2A, B), which indicated that the tobacco promoter (P*rrn*) could successfully drive transgene expression in the kiwifruit plastid. The tobacco P*rrn* promoter was also active in poplar plastids (Okumura et al. 2006; Wu et al. 2019), suggesting that a species-specific promoter may not be required for transgene expression in plastids.

In the six spectinomycin-resistant shoots, four (*Ac*-pQQC7#2–#5) were negative in PCR tests (Fig. 1L–M), and are likely to be spontaneous resistance mutants that arise through acquisition of point mutations in the 16S rRNA gene (Svab and Maliga 1991). Given that the point mutants are antibiotic specific, without conferring cross-resistance, employing spectinomycin and streptomycin double selection could effectively prevent the occurrence of spontaneous mutant events (Bock 2001). This negative frequency is close to that obtained in transplastomic poplar (Okumura et al. 2006; Wu et al. 2019). Notably, in the negative events, shoots were induced directly from leaf explants, whereas green calli were first induced, with subsequent formation of positive shoots (Fig. 1D). The spontaneous mutants associated with using spectinomycin are common in plastid transformation events regenerated from leafy tissue, such as tobacco (Svab and Maliga 1991), potato (Sidorov et al. 1999), tomato (Ruf et al. 2001), and *Lesquerella fendleri* (Skarjinskaia et al. 2003). Especially, the proportion of spontaneous mutants reached 98% (108 out of 110) for *L. fendleri* (Skarjinskaia et al. 2003). In contrast, spontaneous spectinomycin mutants appeared to be much less in plastid transformants regenerated from non-leafy tissues, such as soybean (Dufourmantel et al. 2004) and *Arabidopsis* (Ruf et al. 2019).

Given the increasing interest in utilizing the plastid as a chassis for synthetic biology (Boehm and Bock 2019; Scharff and Bock 2014), the availability of a transplastomic technology will also enable synthetic biology applications in kiwifruit. Although the GFP in leaves of transplastomic kiwifruit reached 2.5% of the total soluble protein (Fig. 2C), this level might decrease in the fruits, due to the relatively low expression of transgenes in non-green plastids, such as chromoplasts in fruits. This obstacle might be overcome through the optimization of chimeric expression elements (promoter-5′ UTR combinations) (Caroca et al. 2013; Chen et al. 2022). For instance, the *rrn16* transcripts exhibit similar expression levels in both leaves and green fruits of ‘Hongyang’ kiwifruit (Chen et al. 2022). In addition, it has been observed that ripe kiwifruit still contain green chloroplasts (Nishiyama et al. 2005), making them ideal candidates for molecular farming and synthetic biology application.

Given the complex inheritance of the plastid genome (paternal dominantly, maternal and biparental subordinately) in kiwifruit (Li et al. 2013), transplastomic plants would also be valuable for studying and identifying plastid genome inheritance patterns. Moreover, paternal inheritance is predominant in kiwifruit (Li et al. 2013) and can be propagated easily by cuttings. Therefore, application of the plastid transformation technique to female kiwifruit can be anticipated to minimize the risks of pollen-mediated gene flow.

## Materials and methods

### Plant materials

The plant materials used in the experiment were derived from the mature fruits of ‘Hongyang’ kiwifruit (*Actinidia chinensis* cv. ‘Hongyang’) collected from the Center of Kiwifruit Breeding, Xianning, Hubei Province, China (Chen et al. 2022). Seeds of the mature fruit were sterilized and inoculated on Murashige-Skoog (MS) medium (Murashige and Skoog 1962), containing 3% sucrose, 0.8% agar, and 0.5 mg/L gibberellin A_3_ (GA_3_). In vitro grown leaves of kiwifruit were used as the explants for plastid transformation.

### GUS assay for optimization of biolistic DNA delivery parameters

To optimize the DNA delivery parameters, transient transformations were performed in which kiwifruit leaves were bombarded with a nuclear expression vector (De Marchis et al. 2009; Sidorov et al. 1999), which carries a *GUS* gene under control of the CaMV *35S* promoter. For GUS staining, the bombarded leaves were vacuum infiltrated for 5 min with freshly-prepared staining buffer (Jefferson 1987). The stained samples were incubated, overnight, at 37 °C in dark and rinsed with 95% ethanol before taking photographs.

### Construction of kiwifruit plastid transformation vectors

The reporter gene *GFP* and selection marker gene (*aadA*) cassettes of kiwifruit plastid transformation vector pYY34 was derived from pYY11, which was similar to pYY12 except for a restriction enzyme site (Wu et al. 2017). The pYY11 was produced by co-transforming the backbone of pYY12 digested by NcoI and XbaI and *GFP* fused with ~ 30-bp homology amplified with prime pair GFP-NcoI-F/GFP-NotI-R (Fig. S2A).

The pYY34 vector was generated by ligating four digested DNA fragments using T4 DNA ligase. These fragments included the backbone obtained by digesting pBluescripII KS(+) with SacI and KpnI, the *GFP* and *aadA* expression cassettes excised from pYY11 with SalI and SpeI (Wu et al. 2017), and the left homologous recombination region (LHRR, 1092 bp) digested with KpnI and SalI and the right homologous recombination region (RHRR, 1185 bp) digested with BlnI and SacI. The corresponding flanking sequences for homologous recombination were obtained by PCR amplification from the kiwifruit chloroplast genome (NCBI access number: NC_026690.1) using primer pairs (KpnI)AcLHRR-F/(SalI)AcLHRR-R and (BlnI)AcRHRR-F/(SacI)AcRHRR-R, respectively. For the construction of pQQC7, the *Cr*P*psbA* and *aadA* fragments were PCR amplified using primer pairs (ApaI)CrPpsbA-F/(g10)CrPpsbA-R and (g10)aadA-F/(SphI)aadA, respectively, using pYY34 as the template. Subsequently, an overlap-extension PCR was performed to obtain a *Cr*P*psbA*-*aadA* fragment separated by the 5′ UTR from *gene10* of bacteriophage T7 (*T7g10*). Finally, both the *Cr*P*psbA*-*aadA* fragment and pYY34 were excised with ApaI/SphI and ligated to generate the pQQC7 (NCBI access number: PP816932; plasmid number: 221601, Addgene; Fig. S2B). The All the primers are listed in Table S2.

### Kiwifruit plastid transformation

Fresh young leaves of the kiwifruit seedlings were placed abaxial side up on AcOsM (agar-solid MS medium, 0.1 M sorbitol, 0.1 M mannitol, 3% sucrose; Fig. 1B) overnight in the dark (Maliga 2012; Wu et al. 2019). Afterwards, gold particles (0.6 μm diameter), coated with plasmid DNA pQQC7, were introduced into the plant cells using a biolistic gun PDS-1000/He (BioRad, USA). Following the biolistic bombardment, the leaf samples were diced into 5 × 5 mm and then placed on AcReMs (agar-solid MS medium, 3% sucrose, and combinations of different hormones; Table S1) containing spectinomycin with abaxial side up (Fig. 1C). When the primary spectinomycin-resistant calli or shoots appeared, they were transferred to AcSmM (agar-solid MS medium supplemented with 3% sucrose, 2 mg/L 6-BA, 0.2 mg/L NAA, and 0.3 mg/L GA_3_) supplemented spectinomycin for further culture. The leaves of these lines were subjected to several additional rounds of regeneration for homoplasmy. The growth conditions for the whole selection procedure are 16 h light 20–25 μE m^−2^ s^−1^ at 25 °C and 8 h dark at 20 °C in a growth chamber. The regenerated shoots were then subcultured to 1/2 MS medium supplemented with 1 mg L^−1^ Indole-3-butyric acid (IBA) and spectinomycin to induce root formation. Finally, the shoots were transferred to soil and grown in standard greenhouse conditions.

### DNA isolation, PCR and Southern blot analyses

Total plant DNA was isolated from leaf samples of wild-type and transplastomic kiwifruit plants using a cetyltrimethylammonium bromide-based extraction method (Clarke 2009). For PCR analysis, the total DNA of wild-type and transplastomic kiwifruit leaves were used as templates with two pairs of primers (GFP-F/GFP-R and psaB-aadA-F/psaB-aadA-R). The PCR results were detected by 1% agarose gel electrophoresis. For Southern blot analysis, DNA samples (7 μg total cellular DNA) were digested with BglII and were then separated by electrophoresis in 1% agarose gels and transferred onto a positively charged nylon membranes (GE Healthcare, USA) by capillary action using the semi-dry transfer method. A 643-bp fragment of the *psaB* gene was amplified by PCR from kiwifruit plastid DNA using primer pair AcpsaB probe-F/AcpsaB probe-R (Table S2) and used as hybridization probe to verify plastid transformation. Labeling of the probe and hybridization were performed with the DIG-High Prime DNA Labeling and Detection Starter Kit II following the manufacturer’s instructions (Roche, Switzerland).

### RNA isolation and northern blot analyses

Total RNA was isolated from fresh leaves using the Quick RNA isolation Kit (Huayueyang Biotechnology, China) and following the manufacturer’s instruction. RNA samples (2 µg total RNA) were denatured and separated by electrophoresis in formaldehyde-containing 1.2% agarose gels. The separated RNA molecules were then transferred from the gel to a positively charged nylon membrane (GE Healthcare, USA) using standard blotting protocols. Gene-specific hybridization probes were prepared by PCR amplification from transformation plasmid. PCR primers and their use are listed in Table S2. Hybridization probes were labeled with DIG using the PCR DIG probe synthesis kit following the manufacturer’s protocol (Roche, Switzerland). RNA blots were hybridized at 68 °C using standard protocols.

### Protein extraction and western blot analyses

Total protein was isolated using a phenol-based extraction method (Cahoon et al. 1992). Protein concentrations were measured with the Easy II Protein Quantitative Kit (TransGen Biotech, China). Samples of 2 µg of total leaf protein and a dilution series of GFP standard protein were separated by electrophoresis in 12% SDS-PAGE gels. The gels were either stained with Coomassie Brilliant Blue R-250 stain (Biyuntian Biotechnology, China) or blotted onto polyvinylidene difluoride (PVDF) membranes (GE Healthcare, USA) using wet transfer for 1.5 h. Membranes were blocked with TBS-T (20 mM Tris–HCl, pH 7.6, 150 mM NaCl and 0.1% Tween 20) containing 5% nonfat milk for 1 h at room temperature, and subsequently incubated with primary antibodies against GFP (1:3000 dilution, ABclonal) for 1.5–2 h at room temperature. Membranes were washed with TBS-T (10 min, 3 times, at room temperature), stained with HRP conjugated anti-rabbit secondary antibody (1:10,000) for 1–1.5 h at room temperature, and again washed with TBS-T (10 min, 3 times, at room temperature). Detection was performed with the enhanced chemiluminescence (ECL, Biosharp, China) kit and the AI600 imager (GE Healthcare, USA).

### Detection of GFP fluorescent signal

Subcellular localization of GFP fluorescence in leaves of wild-type and transplastomic plants was determined by confocal laser-scanning microscopy (LSM 980; Zeiss) using an argon laser for excitation (at 488 nm), a 491–654 nm filter for detection of GFP fluorescence and a 646–728 nm filter for detection of chlorophyll fluorescence.

## Supplementary Information

Below is the link to the electronic supplementary material.

Supplementary file1 (PDF 464 KB)

## Data Availability

All data generated in this study are available in the paper.
