# Supplementary material for: Stable plastid transformation in kiwifruit (Actinidia chinensis)
Source: aBIOTECH. 2024 Nov 28;6(1):72–80. doi: 10.1007/s42994-024-00186-0 (PMC11889296; doi:10.1007/s42994-024-00186-0)
Supplement: Supplementary file 1 — Supplementary file1 (PDF 464 KB) [file 42994_2024_186_MOESM1_ESM.pdf]

Supplementary data to

Stable plastid transformation in kiwifruit (*Actinidia chinensis*)

Qiqi Chen[1#], Yuyong Wu[2,3#], Yanchang Wang[1], Jiang Zhang[2,4*], Shengchun Li[2*]

[a] Key Laboratory of Plant Germplasm Enhancement and Specialty Agriculture, Wuhan Botanical Garden, Chinese Academy of Sciences, Wuhan 430074, China

[b] State Key Laboratory of Biocatalysis and Enzyme Engineering, School of Life Sciences, Hubei University, Wuhan 430062, China

[c] School of Pharmacy, Binzhou Medical University, Yantai 264003, China

[d] Shenzhen Branch, Guangdong Laboratory of Lingnan Modern Agriculture, Key Laboratory of Synthetic Biology, Ministry of Agriculture and Rural Affairs, Agricultural Genomics Institute at Shenzhen, Chinese Academy of Agricultural Sciences, Shenzhen 518000, China.

[#]These authors contributed equally to this work.

[*]Corresponding authors: Shengchun Li (shengchun.li@hubu.edu.cn), Jiang Zhang (zhangjiang@hubu.edu.cn).

This PDF file contains

Fig. S1, S2.

Table S1, S2.

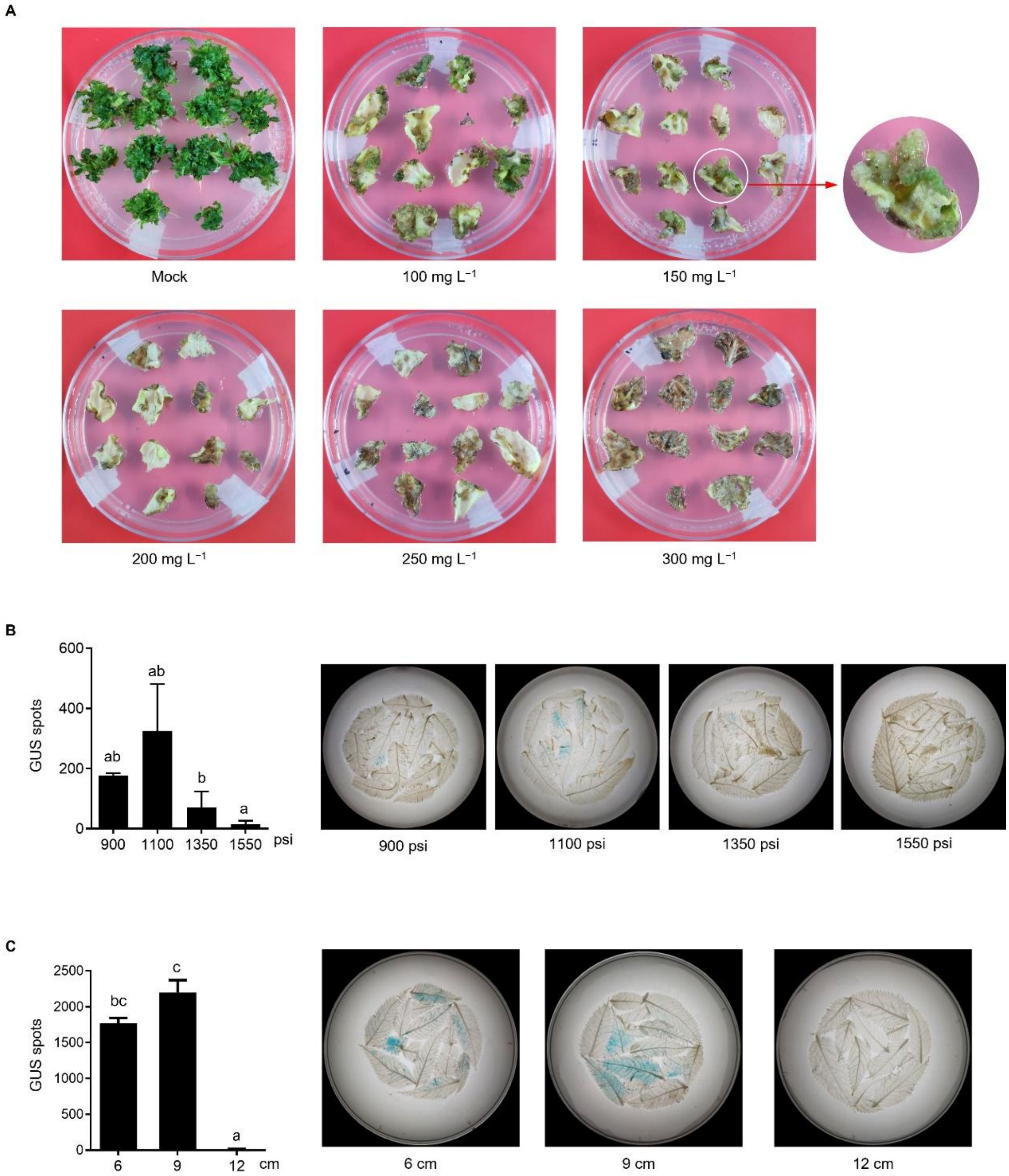

Fig. S1. Optimization of selection and parameters for kiwifruit transformation with gene gun. A Sensitivity test of leaf explants from wild-type kiwifruit to various spectinomycin concentrations regenerated on AcReM3, 60 days after selection. Effects of acceleration pressure (B) and target tissue distance (C) on the results of GUS transient assays. Different letters above the bars indicate the significant of different as determined by Dunnett's T3-test values (p < 0.05).

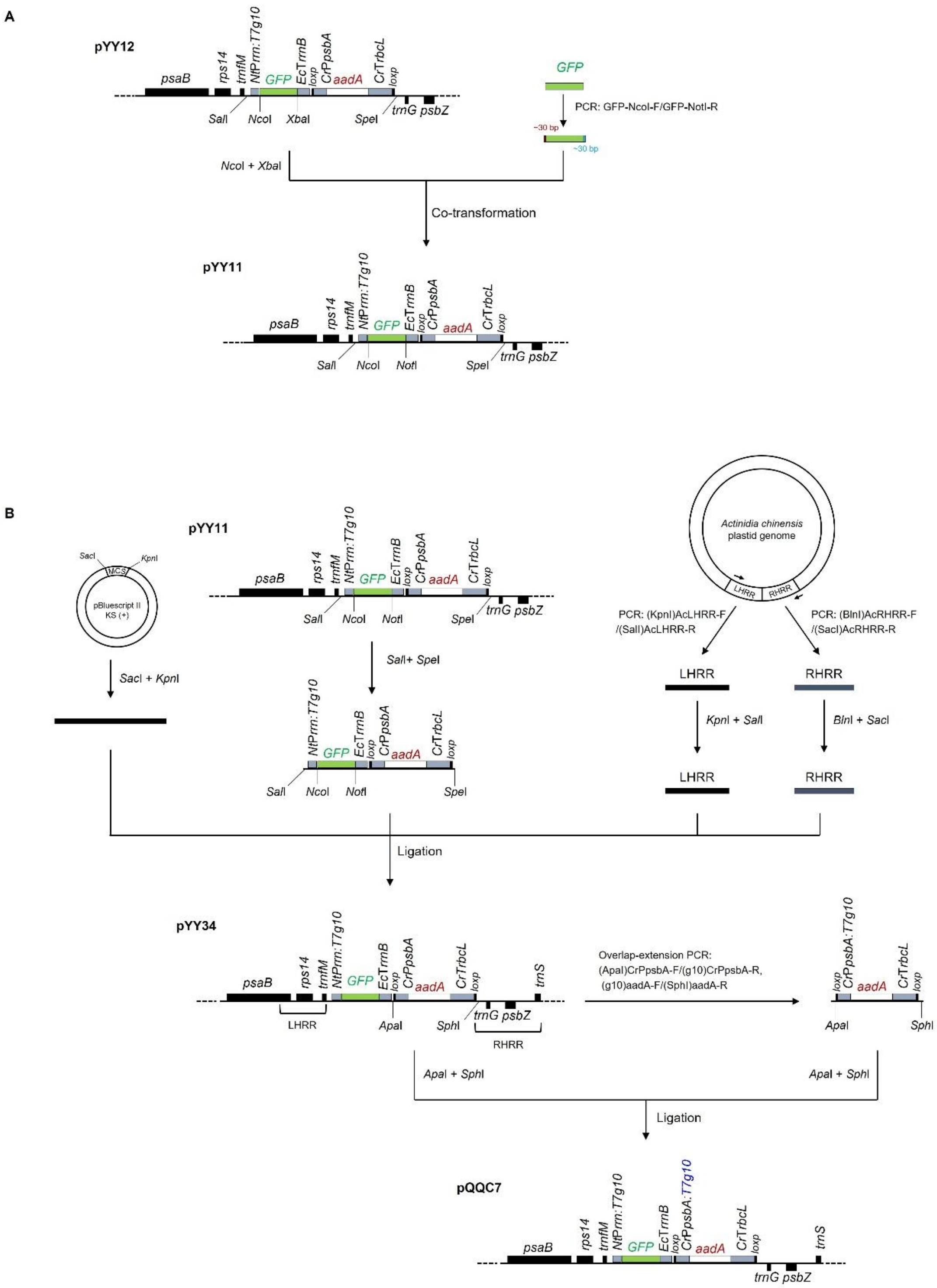

Fig. S2. Generation of kiwifruit plastid transformation pQQC7. A Construction of pYY11 based on pYY12 (Wu et al. 2017). The only difference between them is the restriction site at the end of *GFP*. **B** Outline of the pQQC7 construction. The pYY34 was produced by ligating the backbone of pBluescript II KS (+), *GFP* and *aadA* cassettes from pYY11, LHRR and RHRR from kiwifruit plastid genome. A *T7g10* sequence was inserted between *Cr*P*psbA* and *aadA* gene to generate pQQC7. L(R)HRR, left (right) homologous recombination region.

Table S1. Comparison of different hormone combinations on callus induction and shoot regeneration from leaves of 'Hongyang' kiwifruit.

	Hormone (mg L^{-1})			Number of explants inoculated	Callus induction efficiency (%)	Budding rate (%)	Number of shoots per explant
Medium	TDZ	6-BA	NAA				
Control	-	-	-	48	0	0	0
AcReM1	2	-	1	48	100	100	7.88 ± 2.23 a
AcReM2	2	2	1	48	100	100	8.00 ± 3.13 a
AcReM3	1	2	1	48	100	100	14.13 ± 10.25 b
AcReM4	-	2	1	48	100	100	5.56 ± 3.13 a
AcReM5	-	3	1	48	100	100	4.84 ± 2.59 a

Data were recorded after 60 days of culture for callus induction. Values are mean ± SEM. Different lowercase letters in the same column data indicate significant difference ($P<0.05$).

Table S2 Primers used in this work. Recognition sequences of introduced restriction sites are underlined. The T7 promoter sequence is indicated in italics.

Name	Sequence (5' to 3')	Description and Use
GFP-NotI-F	CTTTAAGAAGGAGATATACCCATGGTGAGTAAAGGAGAAGAACTTTTCACTG	Forward primer for PCR amplification of *GFP*; the primer has 30 base overlapping regions (pYY11 construction).
GFP-NotI-R	AGCCTTTCGTTTTATTTGATGCGGCCGCTCATTGTACAGCTCGTCCATGCC	Reverse primer for PCR amplification of *GFP*; the primer has 30 base overlapping regions (pYY11 construction).
(KpnI)AcLHRR-F	<u>GGTACC</u>CGGTCTGGGCGTGGATGTTC	Forward primer for PCR amplification of left homologous recombination region (LHRR) of kiwifruit plastid genome; introducing a *Kpn*I restriction site (pYY34 construction).
(SalI)AcLHRR-R	<u>GTCGAC</u>CTTAAGCTCGAGCACCTCTTTTCCGTCCATCCC	Reverse primer for PCR amplification of LHRR of kiwifruit plastid genome; introducing a *Sal*I restriction site (pYY34 construction).
(BlnI)AcRHRR-F	<u>CCTAGG</u>GCTAGCCCCGGGGGTAGAACCATTACACTATCACTGCC	Forward primer for PCR amplification of right homologous recombination region (RHRR) of kiwifruit plastid genome; introducing a *Bln*I restriction site (pQQC7 construction).
(SacI)AcRHRR-R	<u>GAGCTC</u>TCTGGCTTGGCTAGGTGGGATA	Reverse primer for PCR amplification of RHRR of kiwifruit plastid genome; introducing a *Sac*I restriction site (pQQC7 construction).
(ApaI)CrPpsbA-F	<u>GGGCCC</u>GGTACCATAACTTCG	Forward primer for PCR amplification of the *Cr*P*psbA* and CrP*psbA-aadA* fragments; introducing a ApaI restriction site (pQQC7 construction).
(g10)CrPpsbA-R	CCGCTTCCCCCATATGTATATCTCCTTCGATGTTAATTTTTTTAAAGTTTTAATTTCTC	Reverse primer for PCR amplification of the *Cr*P*psbA* fragment (pQQC7 construction).
(g10)aadA-F	CTTTAAAAAAATTAACATCGAAGGAGATATACATATGGGGGAAGCGGTGATCGCCGAAG	Forward primer for PCR amplification of the *aadA* fragment; the primer and (g10)CrPpsbA-R primer have 47 base overlapping regions (pQQC7 construction).
(SphI)aadA-R	<u>GCATGC</u>CCTAGACATTATTTGCCGAC	Reverse primer for PCR amplification of the *aadA* and *Cr*P*psbA-aadA* fragments;

		introducing a *Sph*I restriction site (pQQC7 construction).
GFP-F	ATGGTGAGTAAAGGAGAAGAAC	Forward primer for PCR amplification of *GFP* fragment (PCR assay).
GFP-R	TTACTTGTACAGCTCGTCCAT	Reverse primer for PCR amplification of *GFP* fragment (PCR assay).
AcpsaB-aadA-F	GACGAGGCGGTACTTGTGA	Forward primer for PCR amplification of *psaB-aadA* fragment (PCR assay).
AcpsaB-aadA-R	CACTGCGGAGCCGTACAAATGT	Reverse primer for PCR amplification of *psaB-aadA* fragment (PCR assay).
AcpsaB probe-F	AGACCTCCTCCCCATCAAAAGAAAT	Forward primer for PCR amplification of *psaB* probe (Southern blot).
AcpsaB probe-R	TGCACGCGGTTCCAAGTTAATG	Reverse primer for PCR amplification of *psaB* probe (Southern blot).
GFP probe-F	CTTGTTGAATTAGATGGTGATGTTA	Forward primer for PCR amplification of *GFP* probe (northern blot).
GFP-T7 probe-R	*TAATACGACTCACTATAGGG*GCCATGTGTAATCCCAGCAG	Reverse primer for PCR amplification of *GFP* probe, introducing the promoter sequence of T7 RNA polymerase (northern blot).
